# Case Report: Rare cardiovascular characteristics of tuberous sclerosis complex with novel TSC2 variant

**DOI:** 10.3389/fcvm.2024.1464933

**Published:** 2025-01-20

**Authors:** Zhiqin Du, Xiao Ma, Jianhua Li, Fang Yang, Yangfan Guo

**Affiliations:** ^1^ Department of Radiology, Yan'an Hospital Affiliated to Kunming Medical University, Kunming, China; ^2^ Department of Cardiac Surgery, Yan'an Hospital Affiliated to Kunming Medical University, Kunming, China; ^3^ Key Laboratory of Cardiovascular Disease of Yunnan Province, Yan'an Hospital Affiliated to Kunming Medical University, Kunming, China; ^4^ Yunnan Provincial Clinical Research Center for Cardiovascular Disease, Yan'an Hospital Affiliated to Kunming Medical University, Kunming, China; ^5^ Department of Echocardiography and Ultrasound, Yan'an Hospital Affiliated to Kunming Medical University, Kunming, China; ^6^ Department of Pathology, Yan'an Hospital Affiliated to Kunming Medical University, Kunming, China; ^7^ Central Laboratory, Yan'an Hospital Affiliated to Kunming Medical University, Kunming, China; ^8^ Key Laboratory of Tumor Immunological Prevention and Treatment, Yan'an Hospital Affiliated to Kunming Medical University, Kunming, China

**Keywords:** tuberous sclerosis complex, aortic regurgitation, bicuspid aortic valve, imaging diagnosis, genetic diagnosis

## Abstract

**Background:**

Tuberous sclerosis complex (TSC) is a multisystem genetic disorder primarily characterized by the development of benign tumors in multiple organs. While cardiovascular involvement is less common than neurological or renal manifestations, it typically presents with cardiac rhabdomyomas (CRs). The co-occurrence of a bicuspid aortic valve (BAV) with TSC is exceedingly rare.

**Case summary:**

We report the case of a 26-year-old woman with genetically confirmed TSC, harboring a novel pathogenic variant in the *TSC2* gene. Cardiovascular characteristics included a history of heart valve disease, a bicuspid aortic valve, and severe aortic regurgitation. multi-system characteristics of TSC were also presented, affecting skin, brain, lung, kidney, and bone. She underwent aortic valve replacement but experienced postoperative complications, including significant pleural and pericardial effusions requiring drainage and subsequent thoracic duct ligation.

**Conclusion:**

This case expands the clinical spectrum of TSC-associated cardiovascular abnormalities, highlighting the rare association of BAV with this disorder. Our finding emphasizes the importance of considering TSC in individuals presenting with these cardiac features, as well reinforce the critical role of molecular genetic testing in confirming the diagnosis of TSC.

## 1 Introduction

Tuberous Sclerosis Complex (TSC) is an autosomal dominant genetic disorder with an estimated prevalence ranging from 1:6,000 to 1:10,000 (1). TSC presents with a wide spectrum of clinical manifestations involved in multiple organ systems. Over 80% of individuals with TSC exhibit abnormalities of the skin, nervous system, kidneys, lungs, and eyes (2). These can include seizures, developmental delay, skin abnormalities, and benign tumors. Although less frequent than manifestations in other organ systems, cardiovascular abnormalities are still common, with cardiac rhabdomyomas (CRs) present in 47%–67% of individuals with TSC (3). Rarer cardiovascular manifestations include aortic aneurysms (AAs) and myocardial fat foci (4).

Due to this variability in presentation, the diagnosis of TSC requires the detection of clinical findings such as cortical tubers and subependymal nodules in the brain, angiomyolipomas in the kidneys, and lymphangioleiomyomatosis in the lungs, typically through CT or MRI imaging. However, the definitive diagnosis of TSC relies on molecular genetic testing to identify a heterozygous pathogenic variant in a TSC-associated gene (*TSC1* or *TSC2*), regardless of clinical findings (5, 6).

This report describes a patients with genetically confirmed novel pathogenic TSC2 variants who presented cardiac manifestations of TSC. The patient developed severe aortic regurgitation (AR) secondary to BAV. To our knowledge, the association of BAV with TSC is rare.

## 2 Case presentation

**Figure 1:**
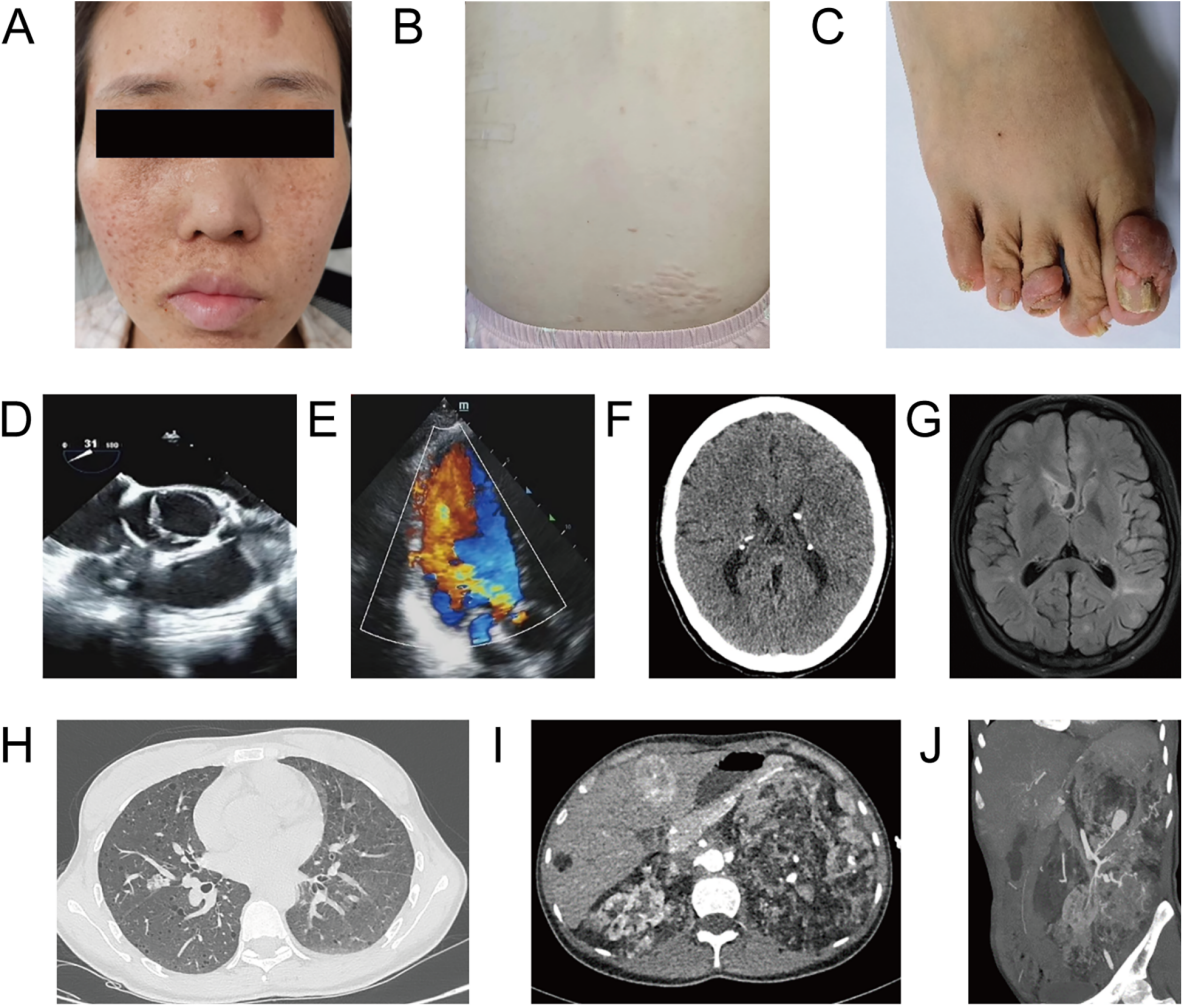
Major clinical and imaging findings in a 26-year-old woman with TSC. **(A)** Facial photograph demonstrates multiple angiofibromas on her face. **(B)** A shagreen patch on the right waist. **(C)** Subungual fibromas affecting toenails. **(D,E)** Echocardiogram showing a BAV with lobe thickening and severe aortic valve insufficiency. **(F)** Head CT demonstrating bilateral calcifications in the lateral ventricles and a nodular calcification. **(G)** Brain T2-weighted FLAIR sequence MR showing multiple cortical and subependymal nodules. **(H)** Chest CT revealing multiple cystic lesions and a well-circumscribed nodule in the right middle lobe. **(I)** Epigastrium postcontrast CT suggesting rapid arterial phase enhancement and delayed washout in liver S4 and fatty nodule without enhancement in S6. Both kidneys are enlarged (left more prominent) with multiple fat-containing lesions. **(J)** MIP image from a CT angiogram of the left kidney demonstrating numerous tortuous, narrowed, and dilated arterioles.

A 26-year-old, unmarried Han Chinese woman residing in rural Yunnan, presented with an eight-year history of heart valve disease. Over the past two years, she had experienced episodes of palpitations and shortness of breath after physical activity, typically lasting several minutes before resolving spontaneously. These episodes had worsened over the preceding two months, prompting her hospitalization. She denied any history of epilepsy, rheumatism, endocarditis, or similar symptoms in her family. Physical examination revealed fibrous plaques on the forehead, multiple facial angiofibroma (Figure 1A), a shagreen patch on the right waist (Figure 1B), and subungual fibromas on both hands and feet (Figure 1C). The apex beat was displaced and diffuse, palpable between the 5th and 6th intercostal spaces of the left midclavicular line, measuring approximately 3 cm × 3 cm. Cardiac percussion revealed cardiomegaly. Heart rate was 84 bpm and regular with a moderate diastolic murmur best heard at the aortic area.

Echocardiography demonstrated a Sievers type 1 LR BAV, with the left valve smaller than the right. Leaflet thickening was observed during systolic phase, and moderate to severe AR was present during diastolic phase (Figure 1D,E). Left ventricular diameter increased and aortic ring diameter was 28 mm. Head CT revealed bilateral calcifications in the lateral ventricles and a 13-mm nodular calcification within the right frontal horn (Figure 1F). Brain T2-weighted FLAIR sequence MRI further delineated multiple cortical and subependymal nodules (Figure 1G). Chest CT demonstrated lymphangioleiomyomatosis in both lungs, characterized by multiple cysts (the largest measuring approximately 22 mm) and a well-circumscribed nodule in the right middle lobe (Figure 1H). Multiple small solid nodules and ground-glass opacities were also present; the largest nodule (20 mm) was in the right middle lobe, outside the segmental bronchus. Hepatic postcontrast CT images suggested a rapid arterial phase enhancement and delayed washout mass in S4, suggestive a hepatic cavernous hemangioma. A fatty nodule without enhancement in S6 was consistent with an AML (Figures 1I). Both kidneys with AMLs were enlarged, with the left more prominent. Left renal CT angiogram revealed multiple tortuous, narrowed, and dilated arterioles within the angiomyolipomas, with several aneurysmal dilations, the largest measuring 27 mm (Figure 1J). Multiple, variably sized sclerotic bone lesions were detected in the skull, spine, and iliac bones.

Multiplex whole-exome sequencing of the patient (proband), her parents, brother, and nieces, using peripheral blood samples, identified that the proband carries a *de novo* heterozygous variant, *TSC2*:c.1841_1865dup. This variant results in a frameshift mutation at codon 623, replacing alanine with leucine, followed by a premature stop codon after one amino acid. According to ACMG guideline, this variant is classified as pathogenic variant (PVS1 + PS2_Moderate + PM2_Supporting), confirming the molecular diagnosis of *TSC2*-associated TSC.

**Figure 2:**
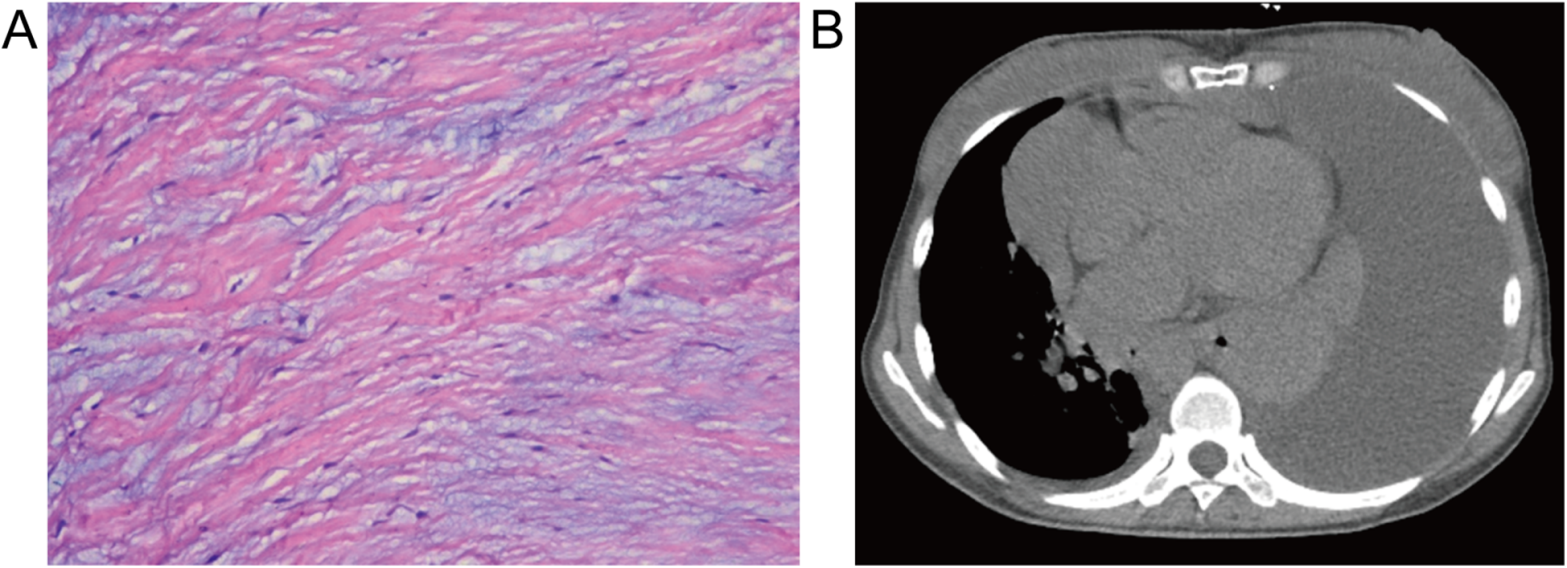
Histopathological and postoperative imaging findings. **(A)** Resected aortic valve demonstrating fibrous tissue hyperplasia with hyaloid and mucoid degeneration (hematoxylin-eosin stain, ×100). **(B)** Postoperative mediastinum CT revealing a large pleural effusion and pericardial effusion in the left thorax and pericardium.

Following the diagnostic workup, the patient underwent routine aortic valve replacement surgery, confirming a BAV with leaflet thickening, severe aortic regurgitation no calcification, no adhesion, and aortic ring dilatation. The diseased valve was replaced with a prosthetic valve. Pathological examination showed fibrous tissue hyperplasia with hyaline and myxoid degeneration (Figure 2A). Chest CT eleven days post-surgery revealed large left-sided pleural and pericardial effusions (Figure 2B), requiring thoracentesis and pericardiocentesis, which drained chylous fluid and hematoma, respectively. The patient's symptoms subsequently improved, and she was discharged. However, she presented again shortly thereafter with a large pleural effusion, requiring thoracic duct ligation. Follow-up chest CT at 10 months showed a small residual pleural effusion.

**Figure 3:**
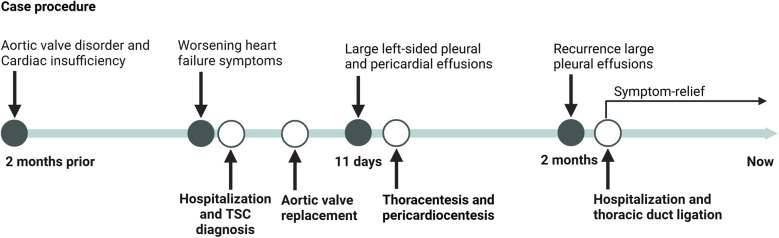
Timeline of diagnosis and treatment for case of TSC.

The timelines of the diagnosis and treatment for patient is shown in Figure 3 Notably, the patient remain clinically stable at the most recent follow-up, with no evidence of disease progression or new complications.

## 3 Discussion

We presented a case of TSC associated with a novel heterozygous pathogenic variant in the *TSC2* gene. Cardiovascular manifestations in this patient led to aortic regurgitation (AR), a relatively rare finding in TSC. TSC is caused by pathogenic variants in either the *TSC1* or *TSC2* gene. *TSC1*, located on chromosome 9q34.13, encodes the protein hamartin, while *TSC2* is located on chromosome 16p13.3 (7) encodes the protein tuberin. These proteins form an intracellular complex that regulates the mechanistic target of rapamycin (mTOR) pathway, a complex signaling network that regulates cell growth, proliferation, and metabolism. Pathogenic variants in *TSC1* or *TSC2* lead to dysregulated cell growth, tumor formation, and tumor progression (8, 9). The majority (two-thirds) of TSC cases arise from *de novo* mutations, with *TSC2* mutations being more common (10).

While CRs are the most common cardiac manifestation of TSC, rarer valvular abnormalities, such as AR can also occur (11). In this case, the echocardiogram demonstrated isolated AR without valvular stenosis. Common etiologies of chronic isolated AR include congenital malformations, rheumatic heart disease, and infective endocarditis. This patient had no history of infective endocarditis or rheumatic heart disease, and echocardiography did not reveal any valvular adhesion or calcification. Furthermore, BAV is a frequent congenital malformation of AR. In this case, the patient exhibited a Sievers type 1 LR BAV with a raphe, supporting a diagnosis of congenital BAV (12). BAV is a common congenital heart defect with a prevalence of 1%–2% (13, 14). While approximately 90% of BAV cases exhibit an autosomal dominant inheritance pattern (15), research suggests a more complex genetic etiology involving multiple genes and environmental factors. Genes implicated in BAV include *NOTCH1* (16), *GATA5* (17), *NOS3* (18), and *TGFBR1/2* (19), which are involved in various aspects of cardiac valve development and extracellular matrix remodeling. For instance, NOTCH signaling is critical for the endothelial-to-mesenchymal transition essential for valve formation (16). Disruptions in these developmental pathways may contribute to BAV and associated abnormalities, such as the aortic root dilatation observed in this case, which can lead to AR. However, whole exome sequencing did not identify any pathogenic or likely pathogenic variants in known BAV-associated genes. This raises the possibility that BAV in this case could be a complication of TSC, rather than an unrelated coincidence. Typically, TSC-associated BAV manifests secondary to CRs, resulting in valvular obstruction or stenosis. However, this case presented BAV without CRs, an exceptionally rare finding that complicates determining the etiology.

Analyzing whether the BAV in this patient is a consequence of TSC, rather than an incidental finding, holds significant clinical value. literature review revealed only one reported case of BAV as a complication of TSC, published in 1976 (20). However, this report lacked a detailed investigation of the underlying cause. Several potential mechanisms could link TSC to BAV. Firstly, the pathogenic variant identified in this case (*TSC2*:c.1841_1865dup, p.Ala623Leufs*2) introduces a premature termination codon, likely resulting in either a truncated *TSC2* protein or degradation of the mRNA transcript through nonsense-mediated decay, ultimately leading to mTOR pathway dysregulation, disrupting cell proliferation, migration, and differentiation during valvulogenesis (21), potentially contributing to BAV formation. Secondly, TSC can disrupt extracellular matrix remodeling, particularly in vascular and valvular tissues, leading to aberrant collagen and elastin deposition (22). This dysregulation may compromise the structural integrity and function of valve leaflets, potentially predisposing individuals to BAV. Finally, hemodynamic alterations due to TSC-associated cardiac abnormalities, such as CRs, could also contribute to BAV (23). Abnormal blood flow patterns and altered hemodynamic stress across the developing valves might disrupt valve development and increase the risk of BAV.

TSC-aneurysms, including arteries of aorta and head/neck, occur twice as frequently in TSC patients (0.74%) compared to the general population (0.35%) (24). Most reported cases involve pediatric patients, according to Dana Cristina Craiu's report (25). A hypothesis suggests developmental defect in the arterial wall or vascular hamartomas disrupting the vasa vasorum may contribute to aneurysm formation (26). Pathological studies of TSC-aneurysm walls demonstrate prominent proliferation of smooth muscle cells in the middle of the aorta may contribute to the disordered elastic layer (25). In this case, the left kidney giant hamartoma complicated numerous tortuous, narrowed, or dilated arterioles. The primary cause is possibly a hemangiohamartoma. It is important to note that even aneurysms smaller than 5 mm can increase the risk of rupture and bleeding when angiomyolipomas exceed 4 cm. Therefore, regular follow-up of these aneurysms is recommended, and embolization therapy should be considered when clinically indicated (27).

## 4 Conclusion

As a multisystem congenital disorder, TSC presents with diverse clinical manifestations across various organ systems. This report highlights the rare finding of BAV and severe AR with TSC, emphasizing the wide phenotypic spectrum of this disorder. While CRs and AAs are more prevalent cardiac manifestations of TSC, this case highlight the importance of considering this diagnosis even in the presence of less common cardiovascular findings. A comprehensive approach incorporating thorough clinical evaluation, multi-organ imaging, and genetic testing is crucial for accurate diagnosis and personalized management of TSC-associated cardiovascular disease. This case report provides novel insights into the complex relationships between pathogenic variants, cardiovascular abnormalities, and clinical management in TSC, and providing a scientific basis for further exploration of the mechanisms underlying TSC-associated cardiovascular disease and the development of individual treatment strategies.

## Data Availability

The raw data supporting the conclusions of this article will be made available by the authors, without undue reservation.
